# Assessment of pain and structure after an intra-articular injection of adalimumab in osteoarthritis of the knee: A case report

**DOI:** 10.1097/MD.0000000000021131

**Published:** 2020-07-10

**Authors:** Abhimanyu Vasudeva, Shiv Lal Yadav, Srishti Nanda, Samantak Sahu

**Affiliations:** a Department of Physical Medicine and Rehabilitation, All India Institute of Medical Sciences (AIIMS), Gorakhpur; b Department of Physical Medicine and Rehabilitation; c Pain research and TMS Laboratory, Department of Physiology, All India Institute of Medical Sciences (AIIMS), New Delhi.

**Keywords:** adalimumab, knee, osteoarthritis, pain

## Abstract

**Introduction:**

Tumor necrosis factor alpha (TNF-α) mediated inflammation has been implicated, in knee osteoarthritis, despite being a predominantly degenerative condition.

**Patient Concerns:**

A 56-year old female, a case of left knee pain not responding to conventional conservative strategies.

**Diagnosis:**

A diagnosis of primary osteoarthritis of the left knee, grade 3 osteoarthritis as per the Kellgren-Lawrence Scale was established.

**Interventions:**

She was administered an intra-articular injection of 10 mg of Adalimumab, a commonly used anti-TNF agent.

**Outcomes:**

The patient was evaluated at baseline, 1 month, 3 months, and at 6 months. There was a marked improvement in pain intensity (visual analog scale) and quality of life, despite no objective change on the parameters seen on ultrasound of the knee.

**Conclusion:**

Injection of adalimumab via the intra-articular route into the knee joint in primary osteoarthritis yields promising results.

**What is Known/What being New**

**Known-** TNF-α plays a pivotal role in pain generation and structural degeneration in osteoarthritis of the knee. Anti-TNF agents such as adalimumab have proven disease modifying efficacy in inflammatory rheumatic diseases making them a part of standard care.
**New-** Anti-TNF agents appear to be effective in controlling pain in osteoarthritis of the knee. They may have disease modifying effects and may halt structural degeneration. Intra-articular route appears to be effective considering osteoarthritis is a more localised pathology compared to inflammatory rheumatic conditions.

## 1 Introduction

Knee osteoarthritis is a leading cause of pain, functional limitations, and subsequent reduction in health-related quality of life. Though it is predominantly a degenerative disease, inflammation and neuropathic pain could also contribute to the clinical presentation. Tumor necrosis factor alpha (TNF-α) has been implicated in both inflammation and neuropathy, by disrupting the pro- and anti-inflammatory homeostasis and stimulating nerve growth factor expression.^[1]^ It has also been implicated in structural disease progression.^[2]^ Moreover, in an animal based study anti-TNF treatment could reverse cartilage degradation.^[3]^ Therefore, anti-TNF agents, such as adalimumab, may be beneficial in controlling TNF-mediated pathogenesis with localized action when administered via an intra-articular route.

## 2 Case report

### 2.1 Subject

A 56-year old female, a homemaker, of body mass index of 23.2 presented to us as a diagnosed case of primary osteoarthritis of the left knee. It was grade 3 osteoarthritis as per the Kellgren-Lawrence Scale. She had received an intra-articular injection of a high molecular weight hyaluronic acid 2 years back which transiently improved pain symptoms. At the end of 1 year from the date of that injection, she received 4 injections of platelet rich plasma (PRP) a month apart each. The last PRP injection was 8 months back. The patient did not have any relief in pain after the PRP injections regimen administered. Throughout the course of the intervention, some prescribed exercises were being performed including strengthening of quadriceps and hamstrings, and stretching of the hamstrings. She was also taking precautionary measures in the activities of daily living. There was, however, very little relief in symptoms. An ultrasound of the knee joint was performed to look for structural changes.^[4]^ Distal femoral cartilage thickness at the mid-point of 2 femoral condyles, synovial vascularity, structure of the medial and lateral menisci (echogenicity of cartilage and extrusion from bony margins) were noted by ultrasound. Prior to the intervention, the distal femoral cartilage thickness was 3.94 mm, there were minimal signs of synovial vascularity as seen by color doppler. Both the medial and lateral meniscal cartilages were hypoechogenic. 52% of total width of the medial meniscal cartilage was extruding from the bony margin, whereas 34% of the lateral meniscal cartilage was extruding from the bony margin. In view of inadequate response after 8 months of the last PRP injection, intra-articular injection of adalimumab was planned after taking written informed consent and clearance from the ethics committee at All India Institute of Medical Sciences (AIIMS), New Delhi.

### 2.2 Intervention

An intra-articular injection of 10 mg of Adalimumab was given under ultrasonography-guidance. The procedure was uneventful and the patient did not have any transient discomfort or complications following injection adalimumab. This was ascertained by asking during the period of observation where the patient was requested to stay back for a few hours after the procedure and subsequently also asked during the first follow-up. The patient was put on Tablet Paracetamol 650 mg as per need as rescue medication and post adalimumab injection pill count was on an average 2 tablets per week.

### 2.3 Outcome measures

**Figure 1:**
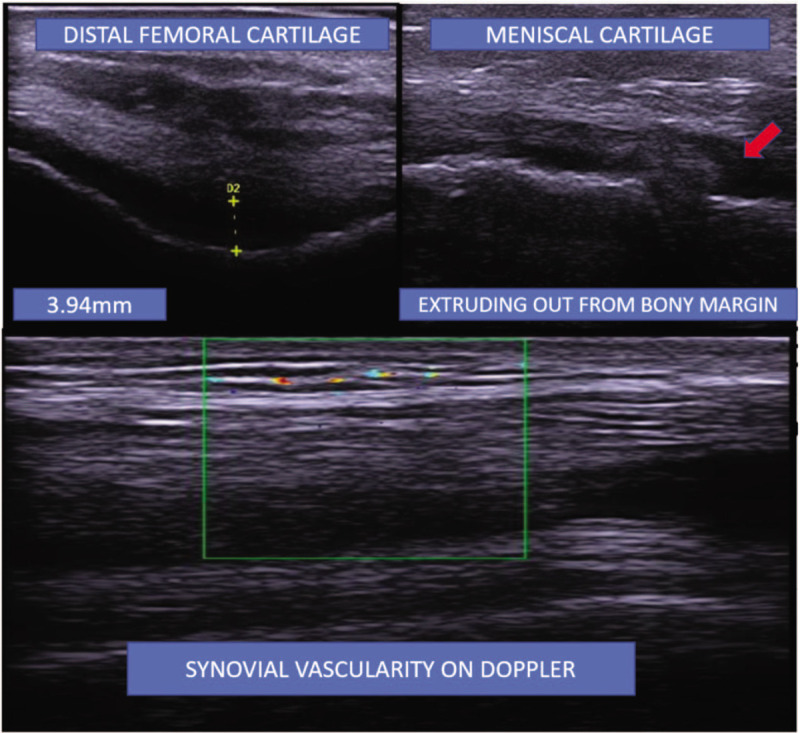
Ultrasonographic evaluation of knee at the baseline. Distal femoral cartilage measured at the mid-point of lateral and medial condyles in the position of knee being maximally flexed.

She was evaluated using the 11-point visual analog scale (VAS) for pain intensity^[5]^ and knee injury & osteoarthritis outcome score (KOOS).^[6]^ The patient was followed up at 1 month, 3 months, and at 6 months for VAS and KOOS. Ultrasonographic examination was repeated at the end of 12 weeks and 6 months (refer to Table 1). Pre- and post-intervention ultrasonographic findings did not show any change despite significant improvement in symptoms (refer to Fig. 1). VAS reduced from 8 (pre-treatment) to 3 at the 1 month follow-up, remained at 3 at the 3 months follow-up and became 4 at the 6 months follow-up. The patient reported that she had noticeable reduction in pain beginning approximately 2 weeks from the date of injection. This was asked at the time of the first follow-up. Total KOOS improved from 32.20 (pre-treatment) to 61.20 at the 1 month follow-up, 60.90 at the 3 months follow-up and 57.50 at the 6 months follow-up (refer to Fig. 1). The patient is under routine care in our OPD and has not complained of any deterioration in symptoms A formal evaluation of VAS, KOOS, and ultrasound examination was however not done after 6 months.

**Table 1:**
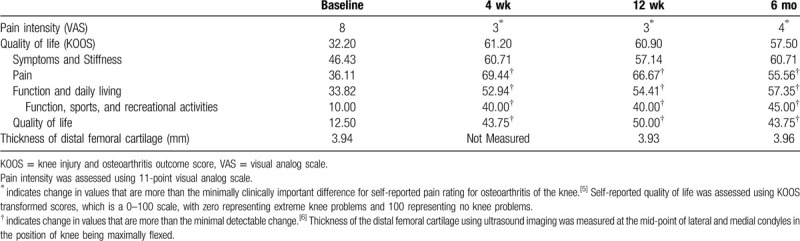
Effect of Adalimumab on outcome measures.

## 3 Discussion

A part of what makes the understanding of the effect of a treatment in osteoarthritis complicated is the inability to pinpoint the pain generator. The cartilage is aneural, and is, therefore, not responsible, at least, directly, in the production of pain, despite its putative role in the pathogenesis.^[7]^ As opposed, there are structures such as the synovium, subchondral bone, periosteum, ligaments, and the joint capsule that are probably the source of pain generation owing to their innervations by nerve endings.^[8]^ This warrants a more comprehensive, yet, feasible approach to look at these very structures whenever searching for the structure-modifying effects of any modality. An ultrasound scan at all the follow-ups may well be the way to go about searching for such structure-modifying effects owing to its inexpensive, accessible, non-invasive, as well as radiation-free visualization of the probable pain generators. However, standardization of grading of such structures on musculoskeletal ultrasound is needed. The marked improvement in symptoms after Injection Adalimumab are unlikely to be due to the residual effects of PRP. Structural improvement or halting the process of degeneration is also unlikely as the patient did not benefit symptomatically at all after the PRP injections.

Dieppe et al^[7]^ suggest a biopsychosocial model of pain in osteoarthritis. A review by Hussain et al^[9]^ argues in favor of a stepwise algorithmic approach, keeping in view the slowly progressive nature of the disease. It may be worthwhile to add adalimumab to this pyramidal approach towards the management of pain in osteoarthritis of the knee in the wake of evidence in favor of inflammation alongside a neuropathic mechanism in the pathogenesis of pain.^[1]^ A 10 mg intraarticular injection of Adalimumab has earlier been used in an randomized controlled trial.^[10]^

It is tempting to adopt an intra-articular route for the administration of adalimumab, however, attempts at understanding this aspect have been made in patients with rheumatoid arthritis and at a higher dose. It was found that both TNF-α and adalimumab can permeate out of the affected joint, albeit, at different rates.^[11]^ TNF-α permeation rate may be different in patients with osteoarthritis and the rate of permeation of adalimumab at a lower dose, specifically, 10 mg may be different. In addition, more research is required to understand its mechanism of action, appropriate dosing, frequency of dosing, means and timing for monitoring structural changes, as well as pharmacokinetic modeling.

## 4 Conclusion

A 10 mg injection of adalimumab via the intra-articular route into the knee joint under ultrasonographic guidance in patients with primary osteoarthritis is promising for the purpose of symptomatic relief despite there being no structure-modifying effects. The findings of the present case are highly encouraging and could serve as a proof-of-concept for a randomized controlled trial.

## Acknowledgments

The authors acknowledge the contributions of DR Asem Rangita Chanu, Assistant professor, Department of Physical Medicine and Rehabilitation for playing a part in the conception of the idea. We also thank IPCA laboratories, India for providing free sample of Adalimumab (ADALIPCA).

## Author contributions

**Conceptualization:** Abhimanyu Vasudeva, Shiv Lal Yadav, Samantak Sahu.

**Data curation:** Abhimanyu Vasudeva, Srishti Nanda, Samantak Sahu.

**Investigation:** Abhimanyu Vasudeva, Samantak Sahu.

**Methodology:** Abhimanyu Vasudeva, Samantak Sahu.

**Project administration:** Abhimanyu Vasudeva, Shiv Lal Yadav, Samantak Sahu.

**Resources:** Shiv Lal Yadav.

**Software:** Abhimanyu Vasudeva, Shiv Lal Yadav, Srishti Nanda, Samantak Sahu.

**Supervision:** Abhimanyu Vasudeva, Shiv Lal Yadav, Srishti Nanda, Samantak Sahu.

**Validation:** Abhimanyu Vasudeva, Shiv Lal Yadav, Srishti Nanda, Samantak Sahu.

**Visualization:** Abhimanyu Vasudeva, Shiv Lal Yadav, Srishti Nanda, Samantak Sahu.

**Writing – original draft:** Abhimanyu Vasudeva, Shiv Lal Yadav, Samantak Sahu.

**Writing – review and editing:** Abhimanyu Vasudeva, Srishti Nanda.

## References

[1] Takano S Uchida K Miyagi M Nerve growth factor regulation by TNF-α and IL-1β in synovial macrophages and fibroblasts in osteoarthritic mice. J Immunol Res 2016;2016:5706359. 27635406 10.1155/2016/5706359 PMC5007361

[2] Goldring MB Osteoarthritis and cartilage: the role of cytokines. Curr Rheumatol Rep 2000;2:459–65. 11123098 10.1007/s11926-000-0021-y

[3] Shealy DJ Wooley PH Emmell E Anti-TNF-alpha antibody allows healing of joint damage in polyarthritic transgenic mice. Arthritis Res 2002;4:R7. 12223110 10.1186/ar430 PMC125301

[4] Backhaus M Burmester GR Gerber T Working group for musculoskeletal ultrasound in the EULAR standing committee on international clinical studies including therapeutic trials. Guidelines for musculoskeletal ultrasound in rheumatology. Ann Rheum Dis 2001;60:641–9. 11406516 10.1136/ard.60.7.641 PMC1753749

[5] Tubach F Ravaud P Baron G Evaluation of clinically relevant states in patient reported outcomes in knee and hip osteoarthritis: the patient acceptable symptom state. Ann Rheum Dis 2005;64:34–7. 15130902 10.1136/ard.2004.023028 PMC1755171

[6] Collins NJ Misra D Felson DT Measures of knee function: International Knee Documentation Committee (IKDC) Subjective Knee Evaluation Form, Knee Injury and Osteoarthritis Outcome Score (KOOS), Knee Injury and Osteoarthritis Outcome Score Physical Function Short Form (KOOS-PS), Knee Outcome Survey Activities of Daily Living Scale (KOS-ADL), Lysholm Knee Scoring Scale, Oxford Knee Score (OKS), Western Ontario and McMaster Universities Osteoarthritis Index (WOMAC), Activity Rating Scale (ARS), and Tegner Activity Score (TAS). Arthritis Care Res (Hoboken) 2011;63 (S11):S208–28. 22588746 10.1002/acr.20632 PMC4336550

[7] Dieppe PA Lohmander LS Pathogenesis and management of pain in osteoarthritis. Lancet 2005;365:965–73. 15766999 10.1016/S0140-6736(05)71086-2

[8] Kidd BL Photiou A Inglis JJ The role of inflammatory mediators on nociception and pain in arthritis. Novartis Found Symp 2004;260:122–33. 15283447

[9] Hussain SM Neilly DW Baliga S Knee osteoarthritis: a review of management options. Scott Med J 2016;61:7–16. 27330013 10.1177/0036933015619588

[10] Wang J Efficacy and safety of adalimumab by intra-articular injection for moderate to severe knee osteoarthritis: an open-label randomized controlled trial. J Int Med Res 2018;46:326–34. 28840750 10.1177/0300060517723182 PMC6011328

[11] Stepensky D Local versus systemic anti-tumour necrosis factor-( effects of adalimumab in rheumatoid arthritis. Clin Pharmacokinet 2012;51:443–55. 22540283 10.2165/11599970-000000000-00000

